# Resveratrol Prevents Oxidative Stress-Induced Senescence and Proliferative Dysfunction by Activating the AMPK-FOXO3 Cascade in Cultured Primary Human Keratinocytes

**DOI:** 10.1371/journal.pone.0115341

**Published:** 2015-02-03

**Authors:** Yasuo Ido, Albert Duranton, Fan Lan, Karen A. Weikel, Lionel Breton, Neil B. Ruderman

**Affiliations:** 1 Diabetes and Metabolism Unit, Boston University Medical Center, Boston University School of Medicine, Boston, Massachusetts, United States of America; 2 L’OREAL Research and Innovation, Aulnay sous bois, France; 3 Endocrinology, Second Affiliated Hospital Chongqing Medical University, Chongqing, China; University of Santiago de Compostela School of Medicine - CIMUS, SPAIN

## Abstract

The aging process is perceived as resulting from a combination of intrinsic factors such as changes in intracellular signaling and extrinsic factors, most notably environmental stressors. In skin, the relationship between intrinsic changes and keratinocyte function is not clearly understood. Previously, we found that increasing the activity of AMP-activated protein kinase (AMPK) suppressed senescence in hydrogen peroxide (H_2_O_2_)-treated human primary keratinocytes, a model of oxidative stress-induced cellular aging. Using this model in the present study, we observed that resveratrol, an agent that increases the activities of both AMPK and sirtuins, ameliorated two age-associated phenotypes: cellular senescence and proliferative dysfunction. In addition, we found that treatment of keratinocytes with Ex527, a specific inhibitor of sirtuin 1 (SIRT1), attenuated the ability of resveratrol to suppress senescence. In keeping with the latter observation, we noted that compared to non-senescent keratinocytes, senescent cells lacked SIRT1. In addition to these effects on H_2_O_2_-induced senescence, resveratrol also prevented the H_2_O_2_-induced decrease in proliferation (as indicated by ^3^H-thymidine incorporation) in the presence of insulin. This effect was abrogated by inhibition of AMPK but not SIRT1. Compared to endothelium, we found that human keratinocytes expressed relatively high levels of Forkhead box O3 (FOXO3), a downstream target of both AMPK and SIRT1. Treatment of keratinocytes with resveratrol transactivated FOXO3 and increased the expression of its target genes including catalase. Resveratrol’s effects on both senescence and proliferation disappeared when FOXO3 was knocked down. Finally, we performed an exploratory study which showed that skin from humans over 50 years old had lower AMPK activity than skin from individuals under age 20. Collectively, these findings suggest that the effects of resveratrol on keratinocyte senescence and proliferation are regulated by the AMPK-FOXO3 pathway and in some situations, but not all, by SIRT1.

## Introduction

Discrete mechanisms that drive healthy aging, rather than disease progression, are largely unknown. At the molecular level, recent research has defined aging as a phenotype caused by epigenetic changes, e.g. DNA methylation, histone modification and aberrant miRNA expression, that increase chronologically in response to intrinsic and extrinsic stimuli [1,2]. Thus, modulating these changes may slow or reverse the appearance of aging phenotypes. In human skin, as in many other tissues, aging is associated with both an increased number of senescent cells (distinguished by their enlarged size and irregular shape) and a reduced capacity for cellular proliferation and differentiation, that contribute to the organ’s reduced regenerative capacity [3,4,5]. Histological analyses of human skin have shown that a younger biological age is associated both with fewer senescent cells and a higher proliferative index [3]. In fact, p16INK4A, an inhibitor of cyclin-dependent kinases and a key regulator of premature senescence, is both a biomarker for human epidermal aging *in vivo* and an inverse correlate of familial longevity [3,6,7].

Consistent with the association between perceived skin age and longevity, both keratinocyte function and regulation of lifespan (in a number of model organisms) share insulin as a common mediator [8,9]. Insulin sensitivity and its intracellular signaling are modulated by AMP-activated protein kinase (AMPK), a fuel-sensing enzyme with diverse physiological roles, as well as sirtuin 1 (SIRT1), a member of the sirtuin family of deacetylases [10,11,12]. Thus, it is not surprising that activation of both AMPK and SIRT1 has been associated with longevity in a number of species [12,13,14]. Yet another factor governing longevity is human forkhead box O3 gene (FOXO3), which encodes an evolutionarily-conserved regulator of insulin signaling that is itself regulated by both AMPK and SIRT1 [15,16,17]. Despite reported interactions between AMPK, SIRT1 and FOXO3, the mechanisms by which they regulate lifespan are not well-understood [18,19,20].

Involvement of AMPK-SIRT1 and FOXO3 in a number of fundamental processes including stress resistance, insulin signaling and longevity suggests that pharmacological modulation of these targets might offer protection against two phenotypes observed in aged sun-protected skin: senescence and impairment of cell renewal. To test this hypothesis, we treated human primary keratinocytes with non-cytotoxic doses of hydrogen peroxide (H_2_O_2_) to mimic the effects of aging (as previously described in cultured keratinocytes [21,22] and *in vivo* in mice and C.elegans [23,24]. We first explored the mechanisms by which resveratrol, an activator of both AMPK and SIRT1 [25,26], regulated two aspects of cellular aging: the development of senescence and the loss of proliferation capacity. In addition, we assessed whether AMPK and FOXO3 played pivotal roles in mediating the effects of resveratrol on these aging characteristics. Finally, we assessed in humans whether decreased AMPK activity is associated with skin aging.

## Materials and Methods

### Cell culture

Primary keratinocytes were freshly isolated from infant penile skin, which was discarded as waste at the time of circumcision. Cells in passages 2–4 that had not reached terminal differentiation were used for this study, as reported previously [22]. They were isolated, selected and cultured in serum-free MCDB-153 medium (Sigma St. Louis, MO USA), which was supplemented with amino acids (50 mg/L of L-histidine, 99 mg/L of L-isoleucine, 14 mg/L of L-methionine, 15 mg/L of L-phenylalanine, 10 mg/L of L-tryptophan, and 14 mg/L of L-tyrosine), and growth factors (2 mg/L bovine pituitary extract, 25 µg/L EGF 5 mg/L insulin, and 50 µg/L PGE1). The medium was changed every other day.

### Procurement of human skin for western blot

Fresh skin tissues were obtained during surgical procedures in which they were discarded as waste. After carefully removing the connective tissue from the skin on ice, the specimens were snap-frozen in liquid nitrogen.

### Ethics Statement

Primary keratinocytes were freshly isolated from infant penile foreskin, which was discarded as waste at the time of circumcision. Collection of these samples at Boston University Medical Center was approved by Boston Medical Center’s Institutional Review Board, as stated in IRB number H23859 entitled “In vitro human flux studies”.

We (L’Oreal Research and Innovation) also obtained skin samples from young and middle-aged humans through BIOalternatives (Gencay, France http://www.bioalternatives.com), a private contract research organization that has a long-standing approved protocol with the University Hospital of Poitiers (Poitiers, France) (n° 1996–010). BIOalternatives received de-identified human skin samples that were discarded as waste during surgical procedures at the University Hospital of Poitiers.

Concerning both the primary keratinocytes and the adult skin samples, the specimens were obtained as discarded waste and therefore exempt from patient consent. Besides gender and age, we do not possess any identifiable information for these samples.

### Resveratrol treatment, senescence and proliferation assays

Resveratrol was obtained from BioMol (Farmingdale, NY) or Enzo (Farmingdale, NY) and dissolved in dimethylsulfoxide (DMSO) at a concentration of 50 mM. Cells were cultured on either 6- or 12-well plates until they reached 40–50% confluence. Sixteen hrs. prior to the treatment (typically 5 days after seeding), the medium was changed to basal medium (growth factor-free). The cells were treated with either DMSO (control), 25 µM resveratrol or 50 µM resveratrol for 30 minutes. Then, 20 µM or 50 µM H_2_O_2_ dissolved in PBS was added and cells were incubated for an additional 2 hrs. The precise concentration of H_2_O_2_ in the 30% stock solution (Sigma) was determined spectrophotometrically by measuring its absorbance and extinction coefficient at 220 nm. Culture medium was then replaced with either fresh growth medium (senescence experiment) or basal medium + 1 nM insulin (proliferation experiment). For proliferation experiments, 13 hrs. after changing the medium, 1 µCi of ^3^H-thymidine (Perkin Elmer, Boston, MA) was added to each well and 3 hrs later, cells were washed twice with 1 ml ice cold PBS. Cells were then washed twice with 1 ml 10% trichloracetic acid (TCA), lysed by 300 µl 0.5N sodium hydroxide (NaOH) +0.1% sodium dodecyl sulfate (SDS) solution and neutralized with 125 µl 1N chlorhydric acid (HCl). After adding 10 ml scintillation solution, ^3^H counts were measured with a scintillation counter (LKB 1219). Some experiments were also repeated by deoxyuridine incorporation methods using Click-it EdU Alexa Fluore 555 imaging kit (Invitrogen). Briefly, 13 hrs after changing the medium, EdU (5-ethyl-2’-deoxyuridine) was added at a final concentration of 10 µM, and 3 hrs later the cells were fixed for fluorescence staining of EdU followed by Hoechst 33342 nuclear staining. Percentage of the cells positive for EdU staining were calculated by assessing 6000–7000 cells. For senescence experiments, 3 days after changing the medium, the cells were fixed for SA-galactosidase (SA-Gal) staining. The latter was done after paraformaldehyde fixation with X-Gal as reported [4]. A total of 300–400 cells in 3–4 wells were evaluated to determine the percentage of SA-Gal positive cells.

### Experiments using recombinant viruses

Recombinant lentiviruses expressing shRNA and GFP protein were prepared as described previously [27,28]. The target sequence for knocking down FOXO3 was GGTGACGCATGTAAATAAA and sequence used to knockdown SIRT1 and AMPK alpha1 were described previously [22,28]. Cells were infected with these recombinant viruses days prior to experiments and infection was monitored by fluorescence microscopy. Titers of these viruses were semi-quantitatively assessed by infecting HEK293T cells. Approximately 20 MOI of the viruses were used.

### Western blot

Western blotting was performed as described previously [22]. The following primary antibodies were used: phospho Thr172 and total AMPK (Cell Signaling, Danvers, MA), FOXO3 (Abcam, Cambridge, MA) and FOXO1, SIRT1 and GAPDH (Santa Cruz Biotechnology, Inc. Santa Cruz, CA)

### FHRE reporter gene assay

FHRE-Luc was obtained from Addgene (Cambridge, MA, #1789) and used as previously described [22]. Briefly, 0.2 µg of plasmid and 0.1 µg of renilla luciferase were co-transfected into keratinocytes grown in 12-well plates using MAX PEI reagent (Polysciences, Warrington, PA). Dual luciferase activity was assessed by using a kit from Promega (Madison, WI).

### mRNA isolation, reverse transcription (RT) and real-time PCR

mRNA was isolated using Trizol. RT reaction was performed using MMLV (Invitrogen Grand Island, NY) with a mixture of dT15 and random hexamer as cDNA templates. Real-time PCR was performed on SmartCycler (Cepheid Sunnyvale, CA) using SYBR qPCR (Clontech Mountain View, CA) reagent with the following primer-pairs: CAT (CATCGCAGTTCGGTTCTCCA, TCCAACGAGATCCCAGTTACCA), BCL2L11 (GCCCTTGTTCCCCCAAATGT, CCTTCTCGGTCACACTCAGAAC), CDKN1B (GCCTCAGAAGACGTCAAACGT, ATGTCCATTCCATGAAGTCAGCG), GADD45A (GGAATTCTCGGCTGGAGAGC, TTCGTACACCCCGACAGTGA), CCNG2 (TCCTGAGCTGCCAACGATAC, GGTGCACTCTTGATCACTGGG), DBB1 (CACTTGCTCTGGGGCTTTCA, CAGACCGCAGTGGCCATAAT), RBL2 (AGCAGTGATAGCAGAAGCCAT, ACCTGTGAGGCGAGTAGGTG), BBC3 (GACCTCAACGCACAGTACGA, GGTAAGGGCAGGAGTCCCAT, this covers isoforms 1, 2, 4).

### Statistics

All experiments were performed at least 3 times. Statistical analysis was performed by two-way anova or one-way anova plus a post-hoc Dunnet test using GraphPad Prism (GraphPad Software). P< 0.05 was considered statistically significant.

## Results

### Senescence

**Fig 1:**
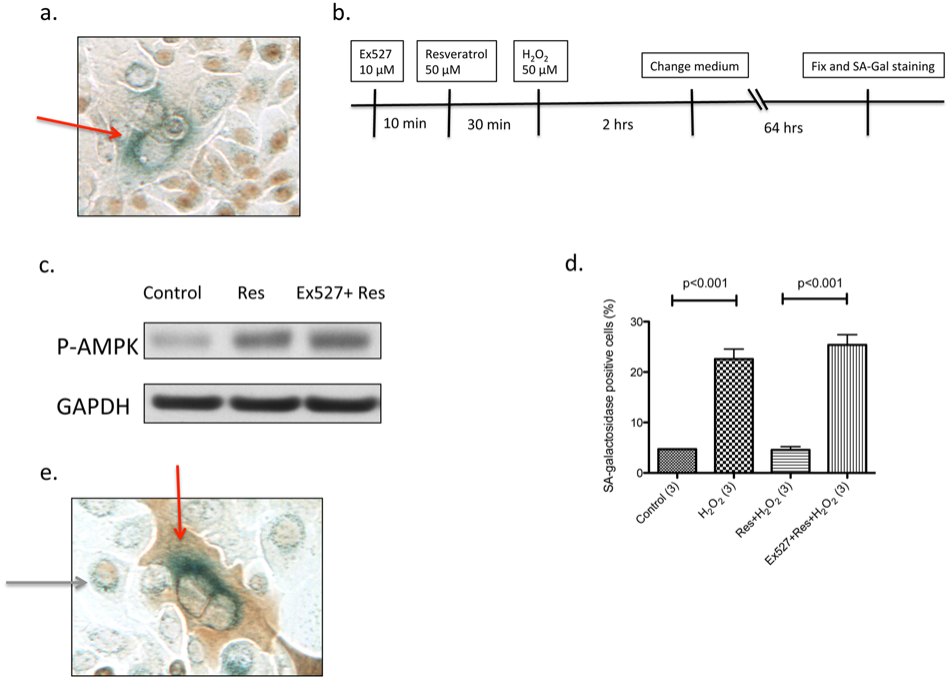
SIRT1 is involved in the attenuation of H_2_O_2_-induced senescence by resveratrol. (a) Human primary keratinocytes (passage 4) showed a number of senescent cells positive for SA-Gal (dark blue-green color). These cells lacked SIRT1 immunostaining (red arrow), which normally appears in the nucleus (see brown color in adjacent cells). (b,c,d) Exposure of human primary keratinocytes to 50 µM H_2_O_2_ induced a 5–6 fold increase in SA-Gal expression. The effect of H_2_O_2_ was largely prevented by pre-treatment with 50 µM resveratrol for 30 min. Addition of Ex527, a SIRT1-specific inhibitor, prior to the addition of resveratrol did not alter AMPK activation but abrogated the effects of resveratrol on SA-Gal staining. For (d), numbers inside parenthesis denote n. (e) SIRT1 was ectopically expressed by recombinant lentivirus, which produced strong staining within the cytosol (red arrow), instead of nuclear staining in normal cells (gray arrow).

**SIRT1 modulates H_2_O_2_-induced senescence in human primary keratinocytes.** We have established that AMPK mediates the prevention of H_2_O_2_-induced senescence by resveratrol in primary keratinocytes [22]. AMPK and SIRT1, another putative target of resveratrol [29], have been reported to regulate one another and to jointly modulate a variety of cellular phenotypes [28,30,31,32]. To determine whether SIRT1 accompanied AMPK in regulating H_2_O_2_-induced senescence, we first evaluated its expression immunohistochemically in senescent keratinocytes. Passaging of keratinocytes (up to passage 4) increased the abundance of senescent cells (stained dark blue-green), and unlike non-senescent cells, they lacked SIRT1 staining (brown) (Fig. 1a, red arrow). To address the role of SIRT1 in the prevention of H_2_O_2_-induced senescence by resveratrol, we treated keratinocytes with 10 µM Ex527, a SIRT1-specific inhibitor, 10 minutes prior to adding 50 µM resveratrol (Fig. 1b). As shown in Fig. 1c, Ex527 had no effect on resveratrol-induced AMPK activation, but it inhibited resveratrol’s ability to prevent senescence (as indicated by H_2_O_2_—induced SA-Gal expression, Fig. 1d). Cells were then infected with lentivirus expressing SIRT1 and stained for SA-Gal and SIRT1. Overexpression of SIRT1 did not change cell shape but surprisingly, staining for SA-Gal and SIRT1 revealed that SIRT1 protein was localized within the cytosol of senesced cells (Fig. 1e red arrow), whereas it was localized in the nucleus of non-senesced cells (Fig. 1e gray arrow).

**Fig 2:**
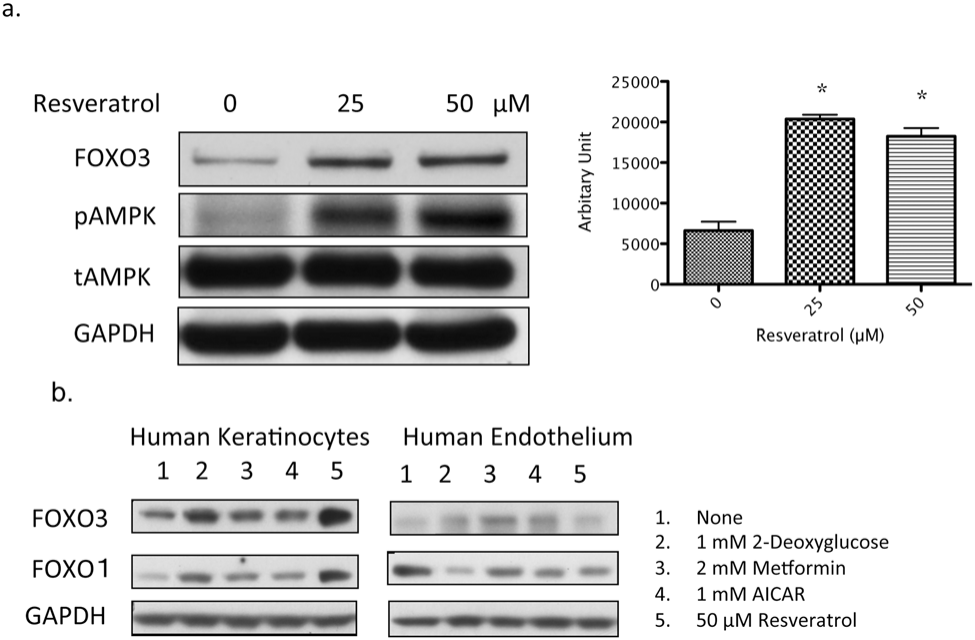
AMPK activation increases FOXO3 protein expression. (a) Keratinocytes grown in a 6-well plate were treated with the indicated concentrations of resveratrol for 30 min. Cells were harvested and subjected to Western blot. 25–50 µM resveratrol induced a 3–fold increase in FOXO3 protein expression. * indicates p<0.05 compared to 0 µM resveratrol treatment. (b) Human keratinocytes and umbilical vein endothelial cells were cultured in 6-well plates until 80% confluence. The cells were then incubated with the indicated reagents for 30 min.-2 hrs. Keratinocytes and endothelial cells were harvested simultaneously for Western blotting. Keratinocyte blotting required only a 30-second exposure for FOXO3 and a 5-minute exposure for FOXO1. Endothelial cell blotting required a 5-minute exposure for FOXO3 and only a 30-second exposure for FOXO1. This suggests that FOXO3 is richer in keratinocytes than in endothelium. Various AMPK activators, in particular 2-deoxyglucose and resveratrol, increased FOXO3 and FOXO1 proteins in keratinocytes. This effect was less apparent in endothelium.

**FOXO3 activation by resveratrol mitigates H_2_O_2_-induced senescence.** The involvement of both AMPK and SIRT1 in resveratrol’s ability to prevent H_2_O_2_-induced senescence suggests that they could work through a common downstream target. One such target is the FOXO family of transcription factors. Increased FOXO activity initiates stress as well as insulin signaling responses [33,34]. Unlike human umbilical vein endothelial cells that we are studying in another line of research; keratinocytes express more FOXO3 than FOXO1 (Fig. 2b). Unexpectedly, resveratrol (Fig. 2a) as well as other AMPK activators (Fig. 2b), acutely (30 min. – 2 hrs.) increased FOXO3 protein levels in keratinocytes. This effect was less apparent in endothelial cells. Immunocytochemistry revealed that the majority of FOXO3 was localized in the cytosol even under basal conditions (growth factors removed overnight) and that this was not altered in response to either H_2_O_2_ (25 or 50 µM) or 50 µM resveratrol+H_2_O_2_ treatment (data not shown). Previously it had been shown that incubation with 500 µM H_2_O_2_ for 30 min. relocalized FOXO3 in the nucleus of MEF cells [35]. We found no such effect in keratinocytes, however (data not shown). Collectively, these findings suggest that the effects of resveratrol and H_2_O_2_ in keratinocytes are mostly mediated by FOXO3 which is already localized in the nucleus.

**Fig 3:**
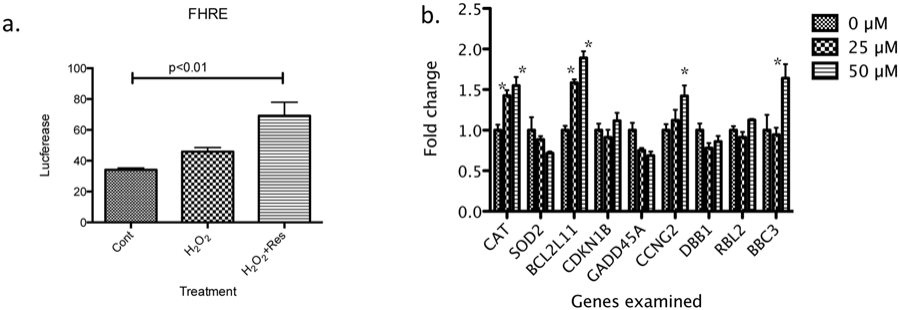
Resveratrol increases FOXO3 transcriptional activity and expression of FOXO3 target genes. (a) FOXO3 transcriptional activity was assessed using the Fork Head Responsive Element (FHRE) reporter gene assay. Cells were grown in 24-well plates and transfected with the reporter plasmid at 40–50% confluence. The assay was performed 48 hrs. later. On the day of the assay, the cells were treated with H_2_O_2_ ± resveratrol for 6 hrs. after which they were harvested and luciferase activity was measured. Resveratrol induced a twofold increase in FOXO3 transcriptional activity. (b) The cells were incubated with the indicated concentrations of resveratrol for 30 minutes and harvested 16 hrs. later. mRNA levels were quantified by real-time PCR. Among the known FOXO3 target genes, expression of catalase (CAT) and BCL2-like 11 (BCL2L11) were significantly increased with 25 µM and 50 µM resveratrol and cell-cycle checkpoint protein cyclin G2 (CCNG2) and proapoptotic protein PUMA (BBC3) were increased by 50 µM resveratrol. * indicates p<0.05 compared to 0 µM resveratrol treatment.

To evaluate whether the resveratrol-induced increase in FOXO3 protein expression in keratinocytes correlates with FOXO3 transactivation, we transfected keratinocytes with a FHRE (Forkhead responsive element) reporter gene plasmid. Six hours after stimulation with H_2_O_2_ or H_2_O_2_+resveratrol, luciferase activity, an indicator of FOXO3 transactivation, was assessed. Although H_2_O_2_ alone tended to increase luciferase expression, the difference was not significant. In contrast, addition of resveratrol together with H_2_O_2_ increased luciferase expression more than two-fold (Fig. 3a). Consistent with this transactivation, we found significant dose-dependent increases in mRNA levels of several FOXO3 target genes, 18 hrs. after exposure to resveratrol in a concentration-dependent fashion. For instance, 25 µM resveratrol upregulated the anti-oxidant catalase (CAT) and apoptosis facilitator protein BCL2-like 11 (BCL2L11) (Fig. 3b). These genes were also upregulated with 50 µM resveratrol, along with two other gene products, cell-cycle checkpoint protein cyclin G2 (CCNG2) and proapoptotic protein PUMA (BBC3) (Fig. 3b). Catalase expression was also upregulated (data not shown).

**Fig 4:**
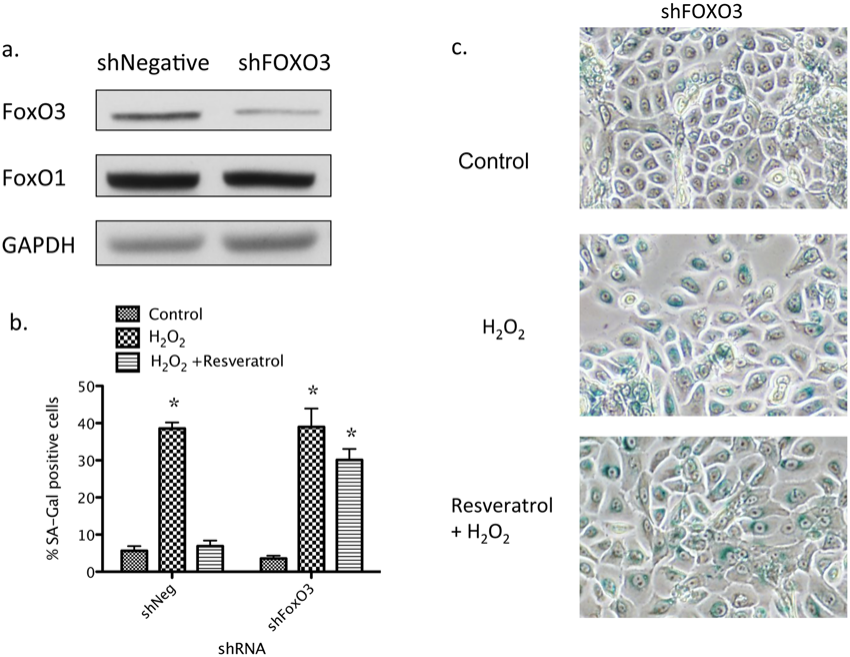
Knockdown of FOXO3 attenuates the suppression of H_2_O_2_-induced senescence by resveratrol. (a) Keratinocytes were cultured in 12-well plates and at 30% confluence, were infected with lentivirus expressing shRNA to non-targeting control (shNegative) and FOXO3 (shFOXO3). Three days later, cells were treated with H_2_O_2_ ± resveratrol, further cultured for an additional 64 hrs. and then fixed with paraformaldehyde for SA-Gal staining, an indicator of senescence. Infection of shFOXO3 lentivirus suppressed FOXO3 expression by 70% but not FOXO1 expression. (b,c) Resveratrol suppressed H_2_O_2_-induced SA-Gal positive staining in shNegative infected cells, but this effect was largely diminished in shFOXO3 infected cells. * indicates p<0.05 compared to control treatment (n = 6).

After establishing that resveratrol activates FOXO3 in primary human keratinocytes, we examined whether FOXO3 function was required for resveratrol’s ability to attenuate H_2_O_2_ -induced senescence. Toward this end, lentivirally expressing shRNA was used to knockdown FOXO3. As shown in Fig. 4a, FOXO3 protein expression but not that of FOXO1 was significantly suppressed 3 days after its infection in keratinocytes. Whereas resveratrol’s suppression of senescence was observed in shRNA negative control (shNeg) infected cells, it was significantly abrogated in FOXO3 shRNA (shFOXO3) infected cells (Fig. 4b and c with magnified images in S1 Fig.). Collectively, this data, along with our previous work [22] indicates that AMPK, SIRT1 and FOXO3 are involved in the attenuation of H_2_O_2_-induced senescence by resveratrol.

### Proliferation

**Fig 5:**
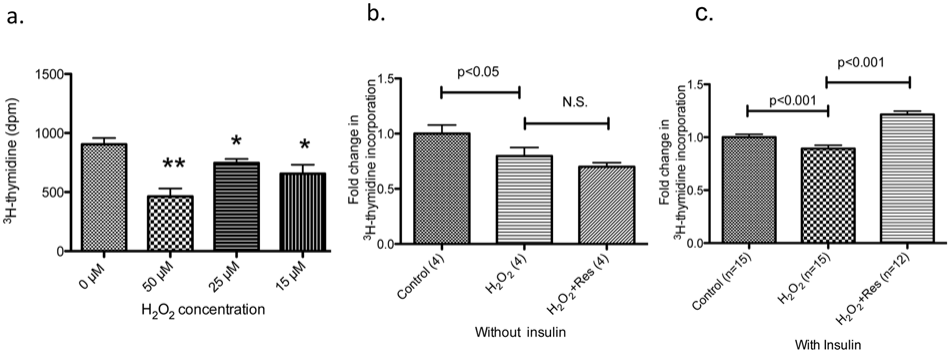
Effects of H_2_O_2_, insulin and resveratrol on ^3^H -thymidine incorporation in human primary keratinocytes. Keratinocytes were grown in 12-well plates until confluency reached 40–60%, at which time they were incubated in growth factor-free basal media overnight. After the indicated treatment, media was changed to basal or basal media containing 1 nM insulin for 16 hrs. ^3^H -thymidine was added for the last 3 hrs. and cells were collected to measure ^3^H using a scintillation counter. (a) As observed previously, incubation with 50 µM H_2_O_2_ for 2 hrs, severely inhibited cell growth and diminished ^3^H—thymidine incorporation, an indicator of cellular proliferation, by about 50%. Lower concentrations of H_2_O_2_ had milder effects. * p<0.05 and ** p<0.01 compared to 0 µM H_2_O_2_ treatment (n = 4). (b,c) Cells were incubated with or without 25 µM resveratrol for 30 minutes prior to treatment with 20 µM H_2_O_2_ for 2 hrs. Media was then changed to a basal medium either without or with 1 nM insulin. Without insulin, resveratrol was not able to prevent H_2_O_2_-mediated suppression of ^3^H -thymidine incorporation (b)_._ In the presence of insulin, resveratrol treatment significantly increased ^3^H -thymidine incorporation by 30% (p<0.001 compared to H_2_O_2_ treatment (c). For (b)-(c), numbers inside parenthesis denote n.

**Resveratrol maintains human keratinocyte proliferation.** The ability of resveratrol to modulate senescence in keratinocytes prompted us to investigate its effects on proliferation, another cellular process affected by aging [36]. Although a number of reports have demonstrated that resveratrol inhibits cellular proliferation [37,38], it has also been shown to increase proliferation in mesenchymal stem cells [39] [40] and to prevent the inhibition of proliferation induced by cyclosporine A [41]. To explore the effect of resveratrol on proliferation in H_2_O_2_-treated keratinocytes, we initially evaluated immunocytochemical staining for the proliferation marker Ki-67 and proliferating cell nuclear antigen (PCNA) [42]. Although cells treated with mitomycin C (an inhibitor of DNA replication) were negatively stained for these markers (as expected), all keratinocytes were positively stained for them 16 hrs after a 2 hr treatment with H_2_O_2_ (data not shown). This indicated that immunostaining was not suitable for measurement of an acute change in proliferation. Therefore we used ^3^H-thymidine incorporation into DNA [43] for this purpose. As shown in Fig. 5a, 2 hr. treatment with 50 µM H_2_O_2_ diminished ^3^H -thymidine incorporation by 50% (Fig. 5a) and by smaller, but statistically significant amounts with 25 µM and 15 µM H_2_O_2_.

Resveratrol treatment alone did not affect the suppression of proliferation mediated by 20 µM H_2_O_2_ (Fig. 5b). However, when keratinocytes were incubated with 1 nM (~166 µU/mL) insulin (which mimics *in vivo* conditions) following the H_2_O_2_ treatment, it significantly increased ^3^H -thymidine incorporation (Fig. 5c). The novel result was also confirmed by a deoxyuridine incorporation assay (S1 Fig.). Consistent with published findings [44], this concentration of insulin (1 nM) by itself had no effect on cell proliferation (Part A in S2 Fig.). In addition, we found that a low dose of H_2_O_2_ (20 µM) was required for resveratrol to exert its effect on proliferation (Part B in S2 Fig. and Fig. 5c). These novel observations suggest that resveratrol and insulin may modulate oxidative stress-induced signaling. To assess this possibility and better understand the roles of insulin and H_2_O_2_ in resveratrol’s actions, all subsequent experiments measuring ^3^H -thymidine incorporation were performed with 1 nM insulin, 20 µM H_2_O_2_ and 25 µM resveratrol.

**Fig 6:**
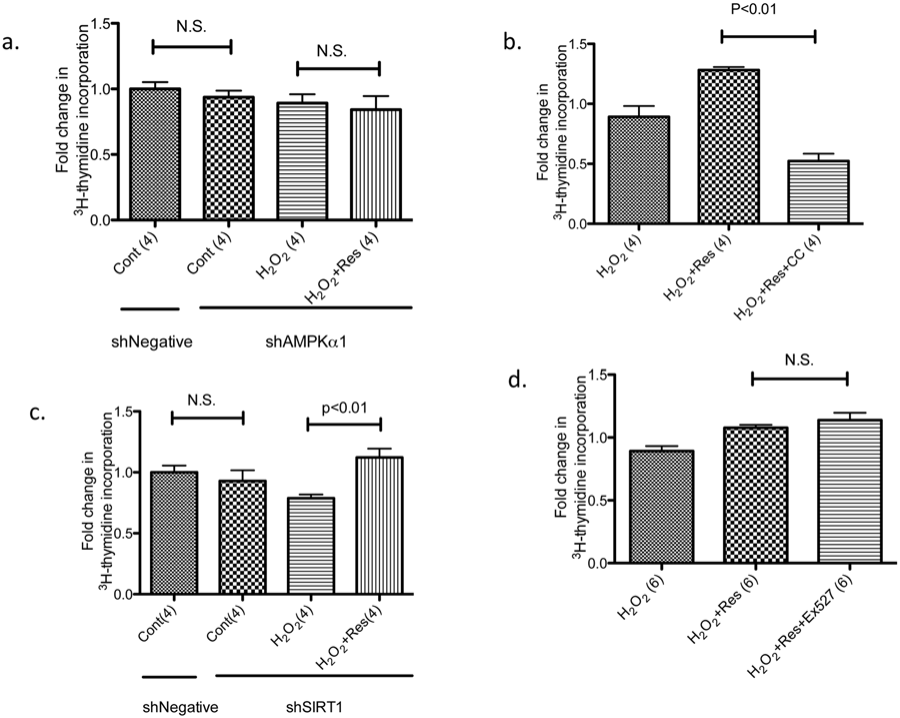
Effects of AMPK knockdown, AMPK inhibitor Compound C, SIRT1 inhibitor Ex527, and SIRT1 knockdown on ^3^H -thymidine incorporation. (a) Keratinocytes were infected with lentivirus vector expressing non-targeting control shRNA (shNegative) or AMPK alpha1 (shAMPKa1). Cells were then treated with 20 µM H_2_O_2_, 1 nM insulin and resveratrol (Res) as described in Fig. 5. Knockdown of AMPK abrogated the effects of resveratrol (which are shown in Fig. 5c). (b) Non-infected cells were treated with H_2_O_2_ and resveratrol as in 6a, but incubated with 1 µg/ml Compound C (CC), an AMPK inhibitor, 30 min. prior to the addition of resveratrol. Compound C inhibited the effects of resveratrol on ^3^H -thymidine incorporation. (c) Keratinocytes were infected with lentivirus vector expressing non-targeting control shRNA (shNegative) or SIRT1 (shSIRT1) that reduced total SIRT1 by about 70%. Knocking down SIRT1 had no effect on resveratrol-induced changes in ^3^H -thymidine incorporation. (d) Non-infected cells were incubated with H_2_O_2_, insulin and resveratrol as in 6a, and 10 µM Ex527 was added 10 min. prior to the addition of resveratrol. Ex527 treatment did not alter ^3^H -thymidine incorporation suggesting that inhibition of SIRT1 did not modulate the effect of resveratrol. For (a) and (c), numbers inside parenthesis denote n.

**Resveratrol-induced cell proliferation is AMPK dependent, but may not be SIRT1 dependent.** We next examined whether this intriguing effect of resveratrol on cell proliferation was dependent on AMPK and SIRT1. To address this question, AMPK inhibitor, Compound C (added to keratinocytes 30 minutes before resveratrol treatment), and AMPK knockdown were employed to inhibit AMPK activity. As described by us previously [22], incubation of keratinocytes with shRNA designed for AMPK alpha1 knocked down 85% of total AMPK protein. AMPK knockdown alone did not affect cell proliferation compared to negative control shRNA; however, it abrogated resveratrol-induced increases in ^3^H -thymidine incorporation (Fig. 6a vs. Fig. 5c). Similar results were obtained in the experiment using Compound C (Fig. 6b). We examined the involvement of SIRT1 in resveratrol-induced proliferation using lentivirus to knockdown SIRT1 [45] [28] and SIRT1 inhibitor Ex527. As shown in Fig. 6c, SIRT1 knockdown did not abrogate the ability of resveratrol to increase ^3^H -thymidine incorporation, nor did Ex527 (Fig. 6d). These results suggest that unlike resveratrol’s attenuation of 50 µM H_2_O_2_-induced keratinocyte senescence, its amelioration of the suppressed keratinocyte proliferation induced by 20 µM H_2_O_2_ is mediated through AMPK but perhaps not SIRT1.

**Fig 7:**
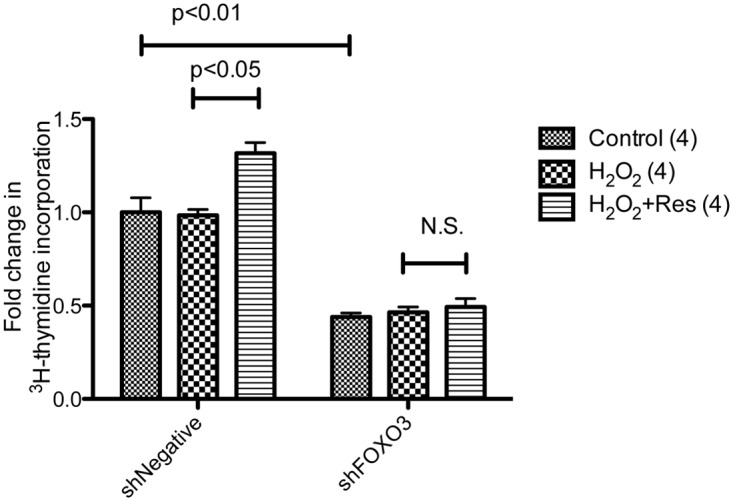
Knockdown of FOXO3 suppresses resveratrol’s effects on ^3^H -thymidine incorporation. Cells were infected with the same lentivirus targeting FOXO3 as described in Fig. 4. Compared to infection with shNegative, infection with shFOXO3 resulted in a 50% reduction in ^3^H-thymidine incorporation. While resveratrol increased ^3^H-thymidine incorporation in shNegative infected cells, the effects were diminished in shFOXO3 infected cells.

**FOXO3 mediates the effects of resveratrol on proliferation in human primary keratinocytes.** The critical roles of insulin and AMPK in resveratrol’s effects on proliferation suggested that similar to senescence, FOXO3 may also regulate proliferation. In keeping with this notion, infection of the keratinocytes with FOXO3 shRNA lentivirus resulted in a significant decrease in the basal rate of ^3^H -thymidine incorporation (Fig. 7). Furthermore, whereas resveratrol was able to increase ^3^H -thymidine incorporation in shNegative-infected cells, it had no effect in shFOXO3-infected cells (Fig. 7). Collectively, these results suggest that FOXO3 is involved in resveratrol’s actions on both senescence prevention and restoration of proliferation.

### Human Study

**Fig 8:**
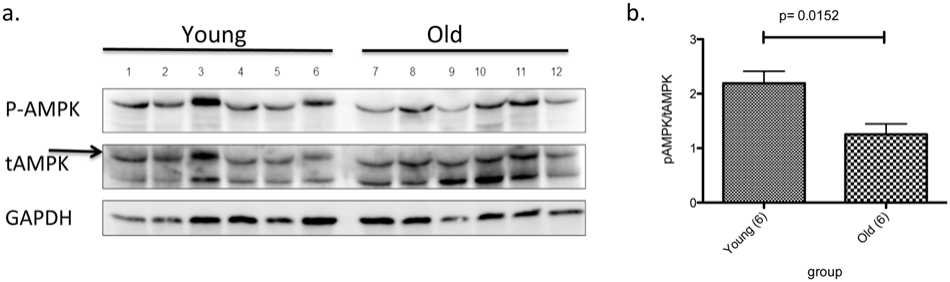
AMPK activation is decreased with aging in human skin. Discarded skin was obtained from patients undergoing a surgical procedure. (a) The samples were processed for Western blot to evaluate AMPK activation levels using p-T172 AMPK and total AMPK (arrow) antibodies. GAPDH was used as a loading control. (b) The ratios of p-T172 to total AMPK were significantly lower in the samples from individuals over 50 years old (n = 6) compared to samples from individuals less than 20 years old (n = 6).

**Basal AMPK activation is decreased in skin from aged individuals.** Our data suggest that decreased AMPK activity may be a mediator of cellular age-related changes in skin. To assess whether it might play such a role in humans, we examined human skin that had been discarded during surgical procedures. As shown in Fig. 8, samples from patients over 50 yrs. old had lower AMPK activity (as illustrated by a lower pAMPK/tAMPK ratio) than skin from younger patients (under 20 yrs. old) Although these data provide support for decreased AMPK activity as an accompaniment of skin aging, the small sample size (n = 6) and incomplete patient characterization necessitate confirmation of such observations in future studies. Likewise, the correlation of these findings with additional manifestation of aging both in skin and other tissues will be of interest.

## Discussion

Human skin is an excellent model in which to investigate aging due to its accessibility for monitoring the effects of both external stresses that contribute to aging phenotypes and of potential “therapies” that prevent them [46,47,48]. Sun exposure is one of the major determinants of skin aging [46,49] due to the distinct effects of its ultraviolet rays; indeed, such extrinsic aging of skin is also called “photo-aging”. On the other hand, differences in perceived skin age are also associated with intrinsic cellular characteristics (e.g. intracellular accumulation of ROS). For example, a better facial appearance at a sun-protected site is thought to reflect the propensity to reach an extreme old age in healthy individuals [47,48,50]. Unlike extrinsic skin aging, the mechanisms that modulate intrinsic skin aging, such as conserved signaling networks that influence longevity, remain unclear.

Resveratrol, AMPK and SIRT1 have been associated with insulin sensitivity and longevity in a variety of model systems [29,51,52]. We confirmed here in a model of intrinsic keratinocyte aging that resveratrol suppresses senescence, a phenotype of cellular aging, through activation of AMPK. Previously, we had shown that resveratrol (AMPK) suppresses H_2_O_2_-induced senescence [22]. In the present study, we establish that SIRT1 is also a key player in keratinocyte senescence. Thus, SIRT1 protein was absent from senescent cells in primary keratinocytes, and forced expression of SIRT1 protein was not localized in the nucleus as typically observed. Rather, it was localized in the cytosol, suggesting that SIRT1 protein does not function normally once keratinocytes become senescent. Increased cytosolic SIRT1 may be indicative of dysfunction of the nuclear import machinery and has been reported to be pro-apoptotic [53]. Normal SIRT1 function has been shown to be necessary for mitochondrial function, e.g. maintaining normal energy levels [54]. These findings, in conjunction with the association between mitochondrial integrity and cellular senescence in skin [23], suggest that SIRT1 may be an important regulator of skin aging *in vivo*.

Epidermal tissue is continuously renewed by keratinocyte stem cells under homeostatic conditions. Functional impairment of these stem cells is thought to contribute to age-related losses in the regenerative capacity of epidermis [55,56]. It has been postulated that this loss in regenerative capacity may be due to changes in the environment in the basal layer of the epidermis [57]. To mimic age-related changes in the epidermal microenvironment, primary keratinocytes from young donors were cultured in a growth factor-free medium. We found that AMPK activation by resveratrol prevented oxidative stress-mediated proliferation impairment in these cells (as indicated by reduced ^3^H -thymidine incorporation) and even increased proliferation relative to untreated cells. Surprisingly, inhibition of SIRT1 did not appear to alter the effects of resveratrol on proliferation in this situation. One explanation for this could be that residual SIRT1 and/or compensation by other sirtuins may overcome the effects of the SIRT1 knockdown on proliferation. Alternatively, the involvement of SIRT1 in mediating resveratrol’s effects on senescence, but not proliferation, may be due to a level of oxidative stress in cells with increased senescence (analyzed 64 hrs post- 50 µM H_2_O_2_) that is different from that of cells with suppressed proliferation (analyzed 16 hrs post-20 µM H_2_O_2_).

FOXO3 is a transcription factor with a diversity of functions; they include control of cell cycle, differentiation, resistance to oxidative stress, apoptosis and inhibition of cellular proliferation in response to stress in human cells [58,59]. We observed that FOXO3 is necessary for the attenuation of senescence and maintenance of proliferation in non-stressed and stressed cells by resveratrol. This finding is consistent with its reported role as a downstream target of both AMPK and SIRT1 [32]. However, the observation that FOXO3, but not SIRT1, is involved in resveratrol’s maintenance of proliferation in the face of oxidative stress is surprising, considering the interaction between SIRT1 and FOXO3 in cellular survival against oxidative stress [60]. These data suggest that although the mechanisms by which resveratrol affects keratinocyte senescence and proliferation may share common mediators, the mediators could act on different pathways.

FOXO3 is at the interface between longevity and tumor suppression and in skin, its upregulation accounts for the increased susceptibility of cells to UVB [61]. FOXO3 has also been noted to protect and preserve haematopoietic stem cell function [62]. In cultured keratinocyte systems, functional stem cells are present along with their multipotent progeny, known as “transit-amplifying cells”[63]. In our model, FOXO3 maintained proliferation, and this is probably by preserving the potential of slow-cycling epidermal stem cells [55], thereby allowing proliferation of a greater number of transit-amplifying cells in the ongoing culture. We may also be observing a role for FOXO3 in proliferation indirectly through its promotion of differentiation, as described by Franco et al. for resveratrol-treated erythroid cells [64]. In addition, insulin, which we observed to be required for the actions of resveratrol on proliferation, has been shown to induce keratinocyte differentiation [65]. Perhaps resveratrol activates FOXO3 in keratinocytes which induces their differentiation into an increased number of transit-amplifying cells.

The involvement of AMPK and FOXO3, two molecules whose activation increases insulin sensitivity [66], in mediating resveratrol’s actions is noteworthy. It suggests that insulin signaling plays a key role in H_2_O_2_-induced proliferation suppression in keratinocytes. Although a physiological dose of insulin did not increase keratinocyte proliferation, resveratrol-induced proliferation required the presence of insulin. This suggests that in keratinocytes, resveratrol may increase insulin sensitivity under conditions of oxidative stress. This novel observation becomes less surprising when one considers the inherent differences in insulin signaling between keratinocytes and other cell types commonly used to study this pathway (e.g. skeletal muscle, adipocytes). In keratinocytes, rather than activating Akt, insulin activates a small GTPase, rac1 [67], and STAT3 [68] which jointly mediate cell mobility and proliferation. Therefore, unlike skeletal muscle cells in which physiological doses of insulin activate Akt and suppress FOXO3, in keratinocytes insulin may not suppress FOXO3. Sufficient FOXO3 activity in these cells may therefore facilitate resveratrol’s effects on proliferation. The role of insulin in resveratrol’s effects on proliferation, and whether IGF1, rather than insulin can activate Akt in keratinocytes are topics for future investigation.

Our results stress the functional importance of AMPK and FOXO3 working in concert with insulin to maintain proliferation in the epidermis. This could be of physiological importance since diabetes is a condition in which insulin signaling is impaired, and it is accompanied by epidermal thinning in experimental rat models *in vivo* [69,70]. Proliferative dysfunction in keratinocytes delays epidermal turnover and is associated with decreased epidermal thickness [11,71, 72]. Epidermal thinning, along with compromised barrier function impairs healing in aged or injured skin. Over 6 million patients in the United States alone (2% of the population) suffer from chronic wounds, and this number is expected to increase with the rapid expansion of elderly, diabetic, and obese populations [73]. Furthermore, given the important roles of FOXO3 in both longevity and insulin sensitivity [74], further exploration of the contribution of this signaling pathway to epidermal aging and longevity is warranted.

As far as we know, this is the first report showing that AMPK activity is diminished in human skin with aging. A number of reports indicate that AMPK activity is lower in several tissues of rodents with high-fat diet-induced insulin resistance or diabetes [75]. However, reports in skeletal muscle of patients with or without the metabolic syndrome are conflicting [10]. We recently demonstrated that AMPK activity in adipose tissues from bariatric surgery patients was significantly lower in individuals with insulin resistance than in subjects of comparable weight who were insulin sensitive [76]. A similar relationship between AMPK and insulin resistance has been observed in patients with Cushing’s syndrome,in whom AMPK activity is lower in visceral adipose tissue than in control subjects [77]. In this regard, our finding that AMPK activity is lowered in skin of individuals over 50 years of age than in individuals under age 20 is consistent with the concept of age-related insulin-resistance. Whether increasing insulin sensitivity helps to prevent skin aging, and whether it is achievable through activation of AMPK remains to be determined.

In conclusion, we have demonstrated for the first time in human primary keratinocytes that resveratrol ameliorates two elements of the aging process: increased senescence and decreased proliferation. Resveratrol’s prevention of senescence is dependent upon AMPK, SIRT1 and FOXO3 whereas its upregulation of cell proliferation is dependent on insulin, AMPK and FOXO3, but not SIRT1 in the experimental setup used here (S3 Fig.). Although the mechanisms which drive these two outcomes may not be identical, the involvement of AMPK and FOXO3 in keratinocyte senescence and proliferation indicates that targeting the AMPK-FOXO3 longevity pathway to increase insulin sensitivity and reduce oxidative stress may potentially attenuate skin aging.

## Supporting Information

S1 Fig Cell proliferation assay assessed by deoxyuridine incorporation. Keratinocytes were treated with the same conditions as shown in Fig 5c. In brief, the cells were treated with/without 25 µM resveratrol followed by 20 µM H_2_O_2_ for 2 hrs. and 1 nM insulin for 16 hrs. The deoxyuridine (EdU) incorporation assay was performed according to the manufacturer’s instructions (see Methods). Deoxyuridine incorporated into the nucleus was stained red (upper right panel) and cell nuclei were stained light blue (right lower panel). The ratio, the number of red stained cells to the number of light blue stained cells, was calculated by counting 6000–7000 cells (a total of 6 fields) to estimate the percent of deoxyuridine positive cells. The result was the virtually identical to the one obtained by ^3^H-thymidine incorporation assay shown In Fig. 5c.
(TIF)

S2 Fig Cell proliferation assay assessed by ^3^H -thymidine incorporation. (a) Keratinocytes were exposed to 0 µM or 20 µM H_2_O_2_ for 2 hrs. and medium was changed to basal or basal + 1 nM insulin (I) for a 16 hr. incubation. This concentration of insulin had no effect on ^3^H -thymidine incorporation. (note: typical growth medium for keratinocytes contains about 500 nM insulin). Numbers inside parenthesis denote n. (b) Keratinocytes were incubated with 0 µM (C) or 25 µM resveratrol (Res) for 30 minutes and the medium was changed to basal or basal + 1 nM insulin (I). In this experiment, H_2_O_2_ treatment was omitted. There was a very small increase in the Res + I treatment as compared to the Res treatment alone but it did not reach statistical significance. Numbers inside parenthesis denote n.
(TIF)

S3 Fig Proposed mediators of resveratrol’s actions on H_2_O_2-_induced senescence and proliferation. Attenuation of H_2_O_2-_induced senescence in keratinocytes by resveratrol requires activation of AMPK, SIRT1 and FOXO3, whereas prevention of oxidative stress-induced proliferative dysfunction by resveratrol requires insulin, AMPK and FOXO3. The role of SIRT1 in the effects of resveratrol on keratinocyte proliferation is not clear.
(TIF)
