# Supplementary figures and images for: Resveratrol Prevents Oxidative Stress-Induced Senescence and Proliferative Dysfunction by Activating the AMPK-FOXO3 Cascade in Cultured Primary Human Keratinocytes
Source: PLoS One. 2015 Feb 3;10(2):e0115341. doi: 10.1371/journal.pone.0115341 (PMC4315597; doi:10.1371/journal.pone.0115341)

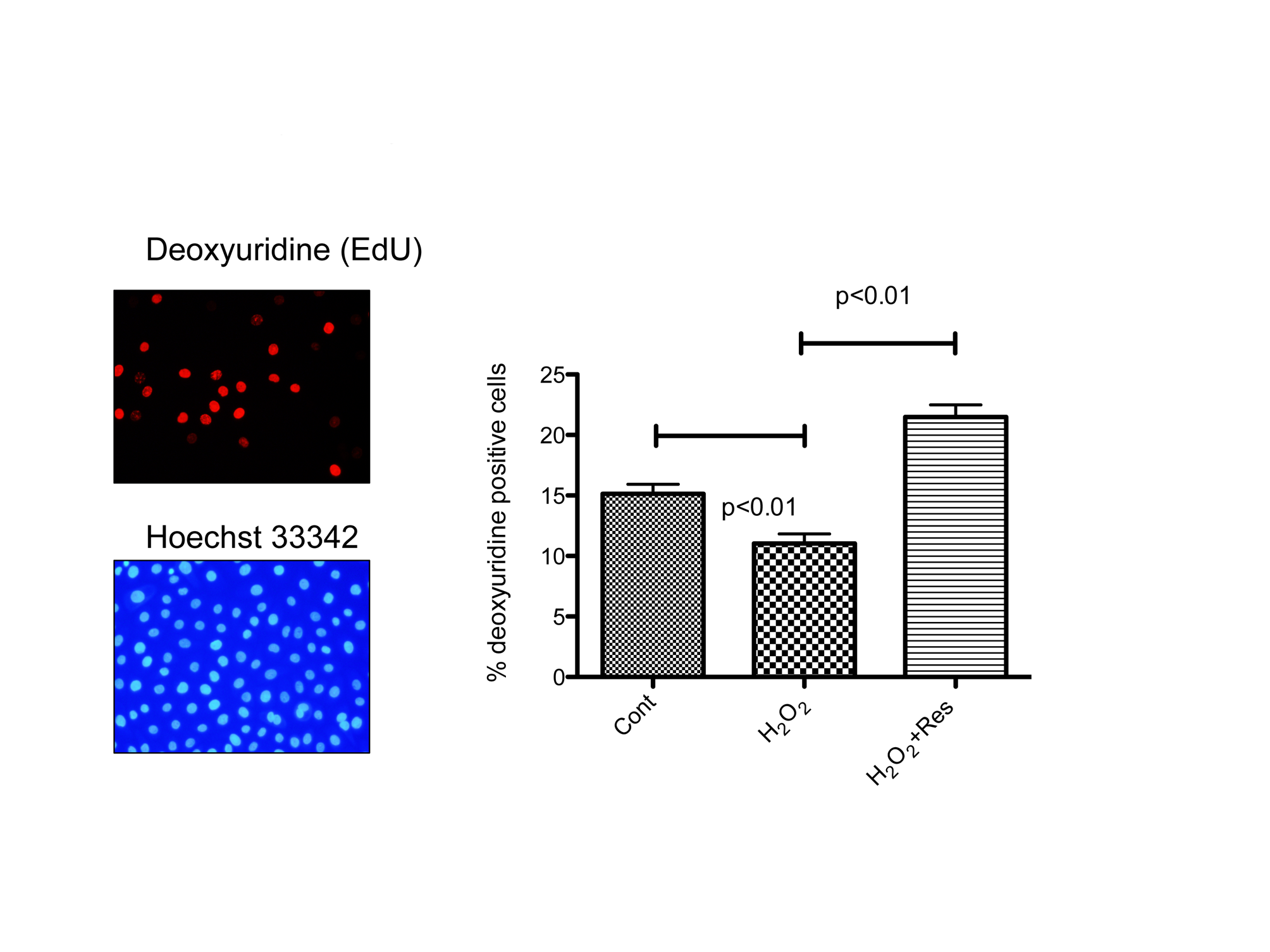

Supplement: S1 Fig — Keratinocytes were treated with the same conditions as shown in Fig 5c. In brief, the cells were treated with/without 25 µM resveratrol followed by 20 µM H2O2 for 2 hrs. and 1 nM insulin for 16 hrs. The deoxyuridine (EdU) incorporation assay was performed according to the manufacturer’s instructions (see Methods). Deoxyuridine incorporated into the nucleus was stained red (upper right panel) and cell nuclei were stained light blue (right lower panel). The ratio, the number of red stained cells to the number of light blue stained cells, was calculated by counting 6000–7000 cells (a total of 6 fields) to estimate the percent of deoxyuridine positive cells. The result was the virtually identical to the one obtained by 3H-thymidine incorporation assay shown In Fig. 5c. (TIF) [file pone.0115341.s001.tif]

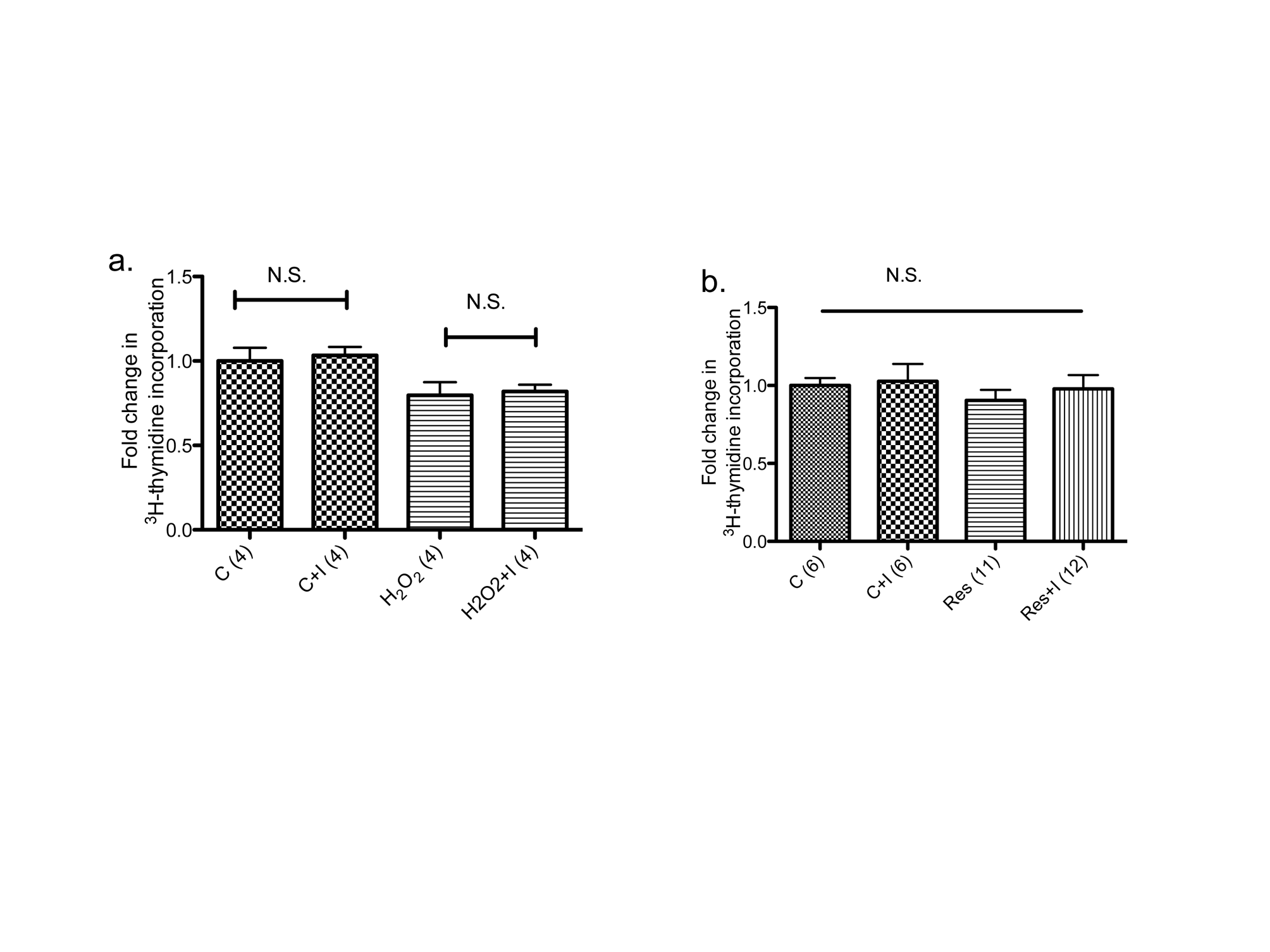

Supplement: S2 Fig — (a) Keratinocytes were exposed to 0 µM or 20 µM H2O2 for 2 hrs. and medium was changed to basal or basal + 1 nM insulin (I) for a 16 hr. incubation. This concentration of insulin had no effect on 3H -thymidine incorporation. (note: typical growth medium for keratinocytes contains about 500 nM insulin). Numbers inside parenthesis denote n. (b) Keratinocytes were incubated with 0 µM (C) or 25 µM resveratrol (Res) for 30 minutes and the medium was changed to basal or basal + 1 nM insulin (I). In this experiment, H2O2 treatment was omitted. There was a very small increase in the Res + I treatment as compared to the Res treatment alone but it did not reach statistical significance. Numbers inside parenthesis denote n. (TIF) [file pone.0115341.s002.tif]

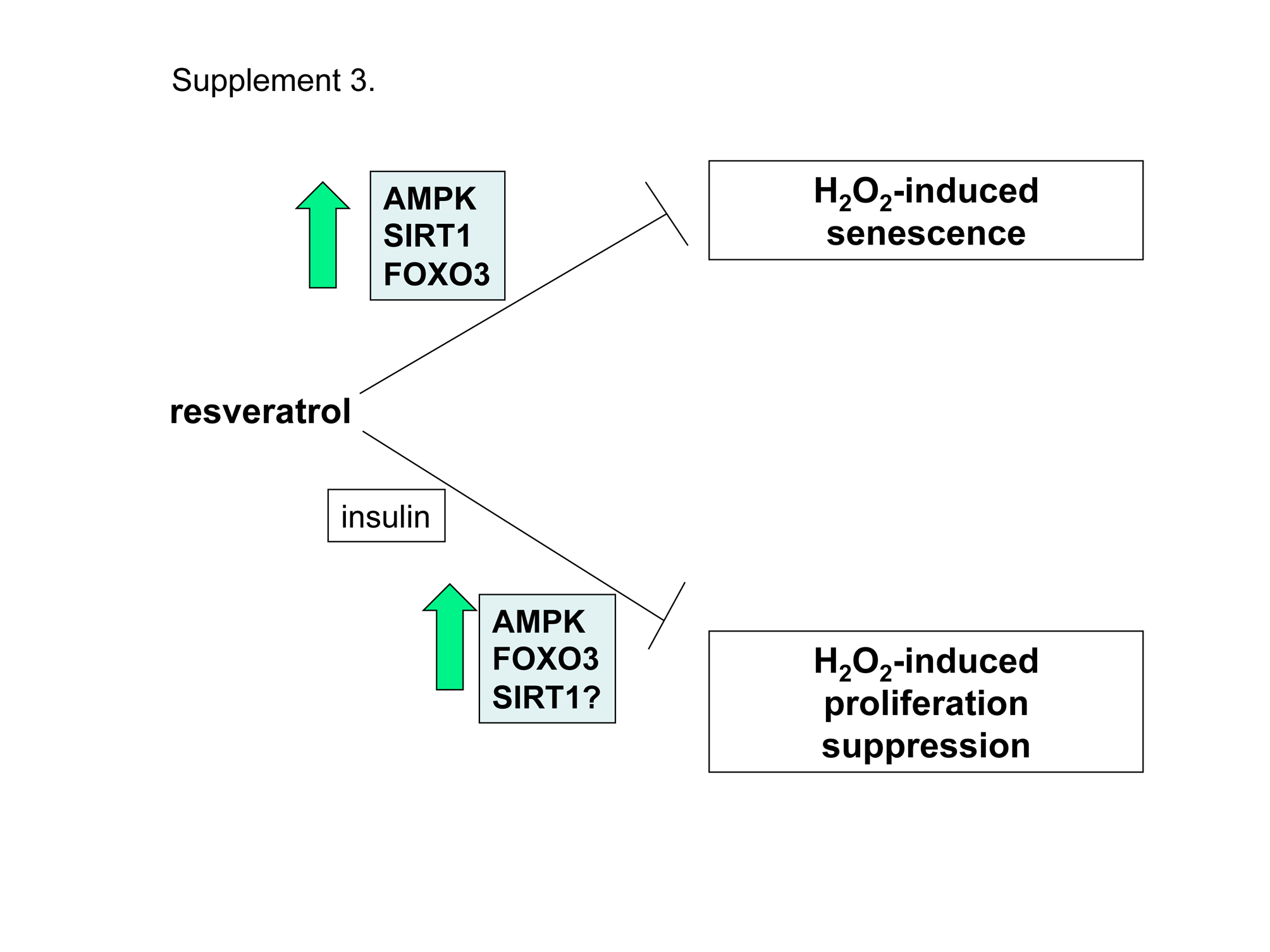

Supplement: S3 Fig — Attenuation of H2O2-induced senescence in keratinocytes by resveratrol requires activation of AMPK, SIRT1 and FOXO3, whereas prevention of oxidative stress-induced proliferative dysfunction by resveratrol requires insulin, AMPK and FOXO3. The role of SIRT1 in the effects of resveratrol on keratinocyte proliferation is not clear. (TIF) [file pone.0115341.s003.tif]
